# Fully Automatic Left Ventricle Segmentation Using Bilateral Lightweight Deep Neural Network

**DOI:** 10.3390/life13010124

**Published:** 2023-01-01

**Authors:** Muhammad Ali Shoaib, Joon Huang Chuah, Raza Ali, Samiappan Dhanalakshmi, Yan Chai Hum, Azira Khalil, Khin Wee Lai

**Affiliations:** 1 Department of Electrical Engineering, Faculty of Engineering, Universiti Malaya, Kuala Lumpur 50603, Malaysia; 2 Faculty of Information and Communication Technology, BUITEMS, Quetta 87300, Pakistan; 3 Department of Electronics and Communication Engineering, SRM Institute of Science and Technology, Kattankulathur 603203, India; 4 Department of Mechatronics and Biomedical Engineering (DMBE), Lee Kong Chian Faculty of Engineering and Science (LKC FES), Universiti Tunku Abdul Rahman (UTAR), Jalan Sungai Long, Bandar Sungai Long, Cheras, Kajang 43000, Malaysia; 5 Faculty of Science and Technology, Universiti Sains Islam Malaysia (USIM), Nilai 71800, Malaysia; 6 Department of Biomedical Engineering, Faculty of Engineering, Universiti Malaya, Kuala Lumpur 50603, Malaysia

**Keywords:** left ventricle, deep learning, spatial features, channel features

## Abstract

The segmentation of the left ventricle (LV) is one of the fundamental procedures that must be performed to obtain quantitative measures of the heart, such as its volume, area, and ejection fraction. In clinical practice, the delineation of LV is still often conducted semi-automatically, leaving it open to operator subjectivity. The automatic LV segmentation from echocardiography images is a challenging task due to poorly defined boundaries and operator dependency. Recent research has demonstrated that deep learning has the capability to employ the segmentation process automatically. However, the well-known state-of-the-art segmentation models still lack in terms of accuracy and speed. This study aims to develop a single-stage lightweight segmentation model that precisely and rapidly segments the LV from 2D echocardiography images. In this research, a backbone network is used to acquire both low-level and high-level features. Two parallel blocks, known as the spatial feature unit and the channel feature unit, are employed for the enhancement and improvement of these features. The refined features are merged by an integrated unit to segment the LV. The performance of the model and the time taken to segment the LV are compared to other established segmentation models, DeepLab, FCN, and Mask RCNN. The model achieved the highest values of the dice similarity index (0.9446), intersection over union (0.8445), and accuracy (0.9742). The evaluation metrics and processing time demonstrate that the proposed model not only provides superior quantitative results but also trains and segments the LV in less time, indicating its improved performance over competing segmentation models.

## 1. Introduction

According to the World Health Organization, cardiovascular diseases (CVDs) are one of the leading causes of death in the world, claiming over 17.9 million lives each year and accounting for approximately 31% of global fatalities [1]. As one of the leading causes of death, cardiovascular disease has drawn considerable attention in medical practice. To assess diagnostic features of the heart, such as ejection fraction and myocardial mass, it is necessary to measure the heart’s volumes. Whereas the precise measurement of the left ventricle (LV) is the most important for figuring out these parameters and diagnosing cardiovascular diseases [2].

Imaging systems and other diagnostic devices such as echocardiography, angiography, magnetic resonance imaging, etc., [3] provide a reliable diagnosis, which is the first step in the control and treatment of CVD. Among these imaging techniques, echocardiography is unquestionably the preferred method for evaluating the heart ventricles. Echocardiography is a user-friendly, reliable, and non-invasive method [4]. It has significant uses in diagnosis and decision-making for numerous CVD. As a result, the echocardiography approach is now the most commonly used approach for evaluating and assessing patients with cardiovascular disease [5,6].

The automatic LV segmentation from echocardiography images can help the sonographer in the detection of diseases. However, semi-automatic or manual annotation is still a part of daily clinical practice. This results in tedious jobs susceptible to intra- and inter-observer variation [7]. In addition, the intrinsic challenges in segmenting echocardiography images, such as low contrast, brightness inhomogeneities, and changes in the speckle pattern, are well established, and several methods [8] are required to eliminate noise to improve the visibility of anatomical features in images.

Recent years have seen the emergence of DL as a fully automated image processing method. The success of the DL model in the medical sector has resulted in a surge of data mining, and the use of DL in a variety of medical areas has particularly gained interest in the field of medical image analysis [9,10,11]. DL has begun to incorporate image registration, detection of the lesion, study of brain function, and image segmentation [12,13,14].

This study, motivated by the effectiveness of DL techniques, proposed a DL technique for LV segmentation from echocardiography images. The following are the main contributions of the proposed method:

- An innovative and lightweight single-stage instant segmentation DL method for fast and accurate LV segmentation from echocardiography images.
- The spatial and contextual data are derived from a single, lightweight backbone model, making the procedure fast.
- The spatial and contextual features are refined simultaneously by two distinct units, which improve the performance of the model.
- An integration unit is used to efficiently combine the features.

The remaining sections of this work are structured as follows: Section 2 provides an overview of the existing LV segmentation architectures. The explanation of each module of the proposed methodology is outlined in Section 3. The experimental setup, datasets, and evaluation matrices are described in Section 4 of the paper. The findings and discussion are presented in Section 5.

## 2. Literature Review

Various techniques, such as the active appearance model, deformable models, and machine learning, have been utilized for LV segmentation and have produced satisfactory results [15,16,17]. Most of these techniques rely on handcrafted features, which may amount to an overly simplistic source of information. In contrast, deep learning (DL) has shown considerable progress in recent studies from the perspective of medical image segmentation techniques [18]. DL models, such as fully convolutional networks (FCN) [19], and U-Net [20], can be referred to as single-stage segmentation model and have attained enormous success in the image segmentation task. The encoder–decoder architectures are employed by both FCN and U-Net. The features are extracted by the encoder, and classification and regression of pixels are performed using those features, whereas the decoder is intended for upsampling to rebuild a semantic segmentation mask according to input image size.

For LV segmentation in CT images, an FCN-based architecture is utilized with pre-trained weights of VGG [21]. Another revised version of FCN with different loss functions is analyzed in [22] and an iterative multi-path FCN (IMFCN) segmentation model is proposed, which segments the LV and RV from MRI images. However, the most significant weakness of FCN is the loss of spatial information. Likewise, U-Net is another widely used DL architecture for LV segmentation [23,24]. The anatomically constrained neural network proposed in [25] is yet another U-Net-based architecture that is employed for LV segmentation from 3D images. The effectiveness of the DL network and the quantity of data needed to train the model for the segmentation of LV by applying the U-Net model were examined by Leclerc et al. [26,27].

In the literature, a few one-stage attention models for medical image segmentation have been proposed [28,29,30]. A model using the attention gate that computes the attention coefficients that scale the input characteristics was proposed by [31]. On top of the U-Net model, a segmentation model with attention gates is developed, and attention gates are transmitted by skip connections on the decoding part of the U-Net. Also, the suggested approach in [32] is based on the attention U-Net segmentation model. An input image pyramid and deeply supervised output are both integrated into the attention U-Net model. This approach segments the desired region and avoids extracting the same low-level characteristics again and again. In [33], a lightweight segmentation model is designed for LV segmentation from echocardiography images. The proposed approach provides a more optimal balance between the number of parameters and segmentation performance. In another study on pediatric echocardiography images, the LV and the left atrium (LA) are segmented using an attention mechanism. The purpose of a spatial path is to extract spatial characteristics, whereas the function of a contextual path is to extract contextual features. Utilizing the fusion model, these two modules’ characteristics are combined [34]. A CNN-based model extracts the spatial data, whereas a backbone model retrieves the contextual information. Although this attention-based LV segmentation achieves high segmentation performance, it may require more time to train and segment since it extracts spatial data using a CNN model and context using another backbone model.

Mask R-CNN [35,36], on the other hand, is a well-known two-stage segmentation model that has achieved an immense amount of success in image segmentation. The CNN is used in the first stage of the two-stage model to filter out some of the proposal boxes, and then the bounding boxes are classified and regressed. The performance of the two-stage model is much better than that of the one-stage model. A comparative study [37] on the performance of Mask RCNN, FCN, and SegNet for LV segmentation indicated that Mask RCNN segmented the LV relatively better than FCN and SegNet [37].

A two-stage model for calculating the clinical indices by segmenting the LV is proposed in [38]. In this study, initially, the LV centers were detected across the cardiac cycle using a Res-circle network. Based on this information, new images with the LV cavity in focus were produced using a cropping approach with pre-defined fixed dimensions. Leclerc et al. [39] presented an attention mechanism intending to improve the segmentation in echocardiography images. The results from the first segmentation network are multiplied by a binary map of the attention model. The U-Net model receives this data as input and uses it to perform the final segmentation. Even though this model decreased the outliers, it did not enhance the accuracy overall. Based on the segmentation performance of the U-Net, [40] proposed a multistage LV segmentation model, referred to as a multistage attention model. In the first phase, U-Net is utilized to suggest the RPN, and in the second phase, regression and segmentation networks execute the segmentation of the LV from echocardiography images.

In summary, models based on convolutional neural networks (CNNs) have shown remarkable improvement over non-DL methods in LV segmentation. The single-stage models are fast enough, but when compared to the two-stage models, the single-stage models’ speed comes at the expense of accuracy when predicting instance classes. Even though two-stage models have improved their level of accuracy, these models are still slow for real-time processing of LV segmentation.

## 3. Methodology

**Figure 1:**
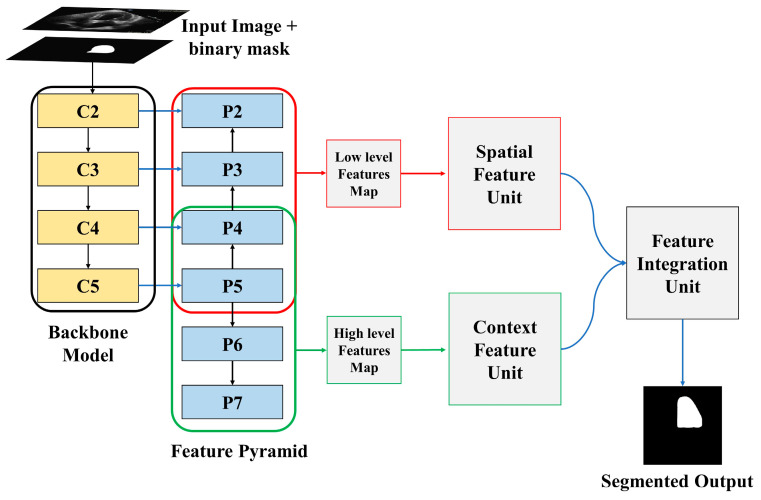
Overview of proposed model.

The proposed LV segmentation model is shown in Figure 1. The training images along with binary masks are transmitted to the backbone model and feature pyramid network, which extracts both low-level and high-level image features using the binary mask of training images. The spatial feature and channel feature modules, respectively, process these low-level and high-level retrieved features. The output of these two modules is then processed by the feature integration unit, which outputs the segmented LV. The following sections explain the entire process in detail.

### 3.1. Backbone Model and Feature Pyramid Network

Residual networks, abbreviated as ResNet, is a traditional neural network used as the backbone for several computer vision applications. ResNet enables the training of hundreds or thousands of layers while still achieving impressive performance. ResNet 50 is used as a backbone model in this study. From a single-scale image of any size, a feature extractor called a feature pyramid network, or FPN, generates correspondingly scaled feature maps at several layers in a convolutional way. It produces a features pyramid that has a similar level of semantic weight regardless of the scale. This objective is simply achieved by building an additional pathway that incorporates high-level features from the upper layers to the lower layers with better resolution, but weaker semantic information.

The input image and binary masks passed through two different channels. The “bottom-up pathway” is the first, while the “top-down pathway” is the second. The spatial sizes must be consistently decreased from bottom to top to generate the pyramid feature maps at different stages of the pyramid. As the image along with the binary mask is passed from bottom to top, the spatial dimension decreases by a factor of one half, achieved by making the stride double. The outputs of the backbone model are denoted by C1, C2, C3, C4, and C5. The output of C5 is employed as the reference set of feature maps for strengthening the top-down path.

The semantically richer feature from higher pyramid levels is translated to higher spatial resolution by the top-down part of FPN by performing the nearest neighbor technique with a factor of 2. Using the skip connection, features of each lateral connection were subsequently merged with equivalent features from the bottom-up path. The bottom-up features undergo 1 × 1 convolution to reduce the channel dimensions, enabling the merging of bottom-up and top-down features. The element-wise addition is used to combine the feature maps from the top-down pathway and the bottom-up pathway. A 3 × 3 convolutional filter is subsequently applied to generate the final feature map. Two more pyramid levels, P6 and P7, are formed in addition to P2 to P5. P7 is created by applying a 3 × 3 filter to P6 whereas P6 is created by applying a 3 × 3 filter to the last convolution layer of the backbone model. The extracted features P2 to P5 are merged and referred to as low-level features, whereas the extracted features P5 to P7 are merged and referred to as high-level features.

### 3.2. Spatial Feature Unit

**Figure 2:**
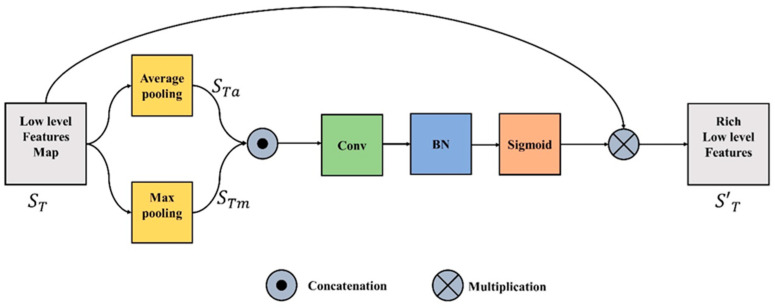
Spatial Feature Unit.

The spatial feature unit (SFU) concentrates on the “where” informative element of the image. The refined and rich spatial features are generated by leveraging the spatial relationship between the features. These spatial features can be obtained by applying a specific operation (${Ꝑ}_{x})$ which takes the input function or tensor $S_{T}\;\epsilon \;S_{T}{}^{H\;\times \;W\;\times \;C}$, where C is a channel plane with a spatial dimension of H x W, and produces a flattened 2D tensor ($S_{T}{}^{H\;\times \;W})$ over the spatial dimension. In the proposed study the two pooling processes (maximum and average) along the channel axis are used to determine the refined spatial features.

(1) $${Ꝑ}_{a}\left(S_{T}{}^{H\times W\times C}\right)\;\to \;S_{Ta}^{H\times W}$$

(2) $${Ꝑ}_{m}\left(S_{T}{}^{H\times W\times C}\right)\;\to \;S_{Tm}^{H\times W}$$

where ${Ꝑ}_{a}$ and ${Ꝑ}_{m}$ in Equations (1) and (2) represent the average and max pooling respectively. To aggregate the channel information, the outputs of Equations (1) and (2) are concatenated. The merged feature descriptor is then fed into a convolutional layer, batch normalization, and the output is passed through sigmoid and transformed to yield the intermediate feature tensor $I_{T}$ represented in Equation (3).

(3) $$S_{T}^{I}=\varsigma \;\left[BN\left\{f\;\left(S_{Ta}^{H\times W}\;ʘ\;S_{Tm}^{H\times W}\right)\right\}\right]\;$$

where ς is the sigmoid operator, BN is batch normalization, f is the convolution operation and ʘ represents the concatenation operation. The final refined spatial features ${S^{\prime }}_{T}$ is produced by multiplying the input feature, $S_{T}$, with intermediate spatial tensor $S_{T}^{I}$, as represented in Equation (4). Figure 2 illustrates the entire process.

(4) $${S^{\prime }}_{T}=S_{T}\times S_{T}^{I}$$

### 3.3. Channel Feature Unit

The focus of the channel features unit (CFU) is on “what” is significant in an input image since each channel of a feature map is thought of as a feature detector. The output of this unit is also known as channel attention and is attained by compressing the spatial dimension of the input feature map. Input channel feature or tensor $C_{T}\epsilon \;C_{T}{}^{H\times W\times C}$ is pooled using two different pooling operations, maximum and average pooling, unlike the spatial module, where the output is a 3D tensor as represented in Equations (5) and (6).

(5) $${Ꝑ}_{a}\left(C_{T}{}^{H\times W\times C}\right)\;\to \;C_{Ta}^{H\times W\times C}$$

(6) $${Ꝑ}_{m}\left(C_{T}{}^{H\times W\times C}\right)\;\to \;C_{Tm}^{H\times W\times C}$$

The maximum and average pooling not only successfully learns the extent of the desired object but also acquires crucial information about the object’s specific characteristics to estimate finer channel-wise attention. The $C_{Ta}^{H\times W\times C}$ and $C_{Tm}^{H\times W\times C}$ are fed to a multi-layer perceptron with one hidden layer. This MLP is simply a fully connected layer and the output generated is shown in Equations (7) and (8).

(7) $$\mathrm{MLP}\left(C_{T}{}^{H\times W\times C}\right)=C_{Ta}^{1\times 1\times C}$$

(8) $$MLP\left(C_{T}{}^{H\times W\times C}\right)=C_{Tm}^{1\times 1\times C}$$

These two outputs are added up element by element, the reshaped summed tensor and sigmoid are applied on the output to generate the intermediate channel feature tensor $C_{T}^{I}$ represented in Equation (9).

(9) $$C_{T}^{I}=\varsigma \;\left[reshape\left(C_{Ta}^{1\times 1\times C}+C_{Tm}^{1\times 1\times C}\right)\right]$$

**Figure 3:**
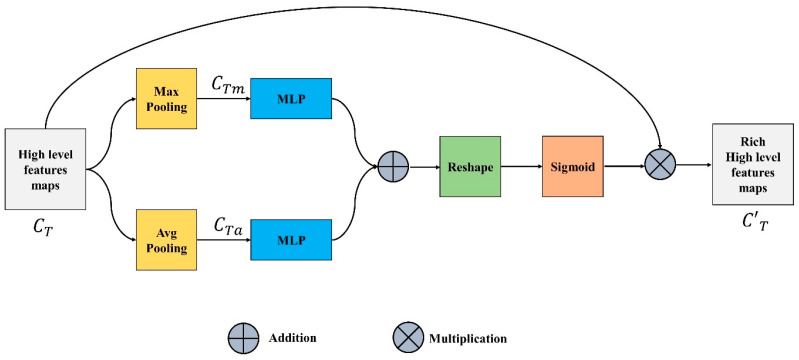
Channel Feature Unit.

Equation (10) illustrates that the refined channel feature ${C^{\prime }}_{T}$ is produced by multiplying the input feature, $C_{T}$ with $C_{T}^{I}$. Figure 3 depicts the complete CFU.

(10) $${C^{\prime }}_{T}=C_{T}\times C_{T}^{I}$$

### 3.4. Feature Integration Unit

There is a distinction in the level of feature representation between the two feature modules. Most of the spatial data acquired by the spatial path incorporate rich detailed information, while the output feature of the channel path includes primarily context information; therefore, directly combining or concatenating low-level and high-level features would inevitably lead to the degradation of information.

Because of this, we integrate low-level and high-level features using the feature integration unit (FIU), which properly merges various features comprehensively. To extract the most important pixel information from high-level features, global average pooling is deployed. Next, convolution, ReLU, and sigmoid functions are applied to $F_{H}$ to acquire the feature indicator $F_{H}$ shown in Equation (11). This $F_{H}$ acts as a guide for low-level features.

(11) $$F_{H}=\varsigma \left[conv\;\{{Ꝑ}_{ga}({C^{\prime }}_{T}\right)\}]$$

Meanwhile, convolution operation and batch normalization are applied on low-level features to adjust and normalize the scale of channels and $F_{L}$ is generated, represented in Equation (12).

(12) $$F_{L}=conv[BN({C^{\prime }}_{T})]$$

$F_{H}$ is multiplied with $F_{L}$ and finally is added to the result of multiplication to obtain the segmented output $O_{s}$ as demonstrated in Equation (13).

(13) $$O_{s}={C^{\prime }}_{T}+(F_{H}\times F_{L})$$

**Figure 4:**
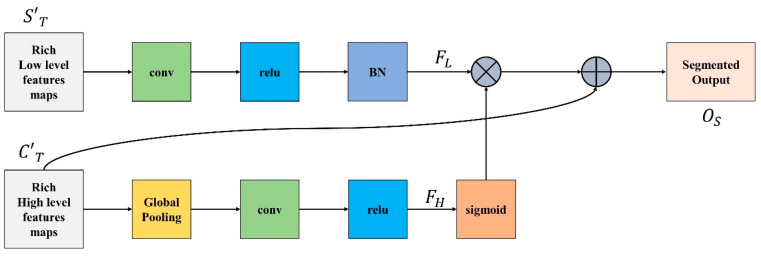
Feature Integration Unit.

Figure 4 demonstrate the complete FIU process.

### 3.5. Loss Function

In DL-based segmentation techniques, the loss function plays a critical role. A loss function is used to calculate how well the current output matches the target during training. Various segmentation tasks have proposed different loss functions. Dice is a loss function belonging to the class of region-based loss functions, which seek to reduce the mismatch or maximize the overlap areas between the ground-truth G and the anticipated segmentation S. In this study, the dice function is used as a loss function and is mentioned in Equation (14).

(14) $$Loss=1-\frac{2\sum_{k=1}^{N}\sum_{l=1}^{M}g_{l}^{k}s_{l}^{k}}{\sum_{k=1}^{N}\sum_{l=1}^{M}g_{l}^{k}+\sum_{k=1}^{N}\sum_{l=1}^{M}s_{l}^{k}}$$

## 4. Experiments

In this section, the dataset used for both model training and testing is presented in detail. In addition, the hardware, hyperparameters, and evaluation metrics used to analyze the performance of the DL models are described.

### 4.1. Dataset

Data for this study was acquired from the National Heart Institute in Kuala Lumpur, Malaysia. It consists of 6000 two-dimensional images of apical four-chamber echocardiography images. These images were taken under protocol number RD5/04/15, which was authorized by Kuala Lumpur, Malaysia’s National Heart Institute Research Ethics Board. The 2-dimensional echocardiogram was performed using a Philips IE33 ultrasound system equipped with an S5-1 (1.0–3.0 MHz) transducer. Every image had a dimension of 800 by 600 pixels, a resolution of 0.3 mm by 0.3 mm, with a frame rate ranging from 30 to 100 Hz. The irrelevant background was cropped out of all images, and the size was reduced to 512 × 512. The dataset is divided into three sections: 70% is used for training, 15% is used for testing, and the remaining 15% is used for validation. The binary masks are generated from the labelled LV. The labelled images were verified by medical professionals.

### 4.2. Network Training

A workstation with a Dell Core i7 Xeon E5-2620 CPU and an 11 GB Nvidia GeForce GTX 1080Ti GPU is used to train the network. An optimizer based on stochastic gradient descent with 0.9 momentum is chosen to be used during the training process. The initial learning rate is set to 10-4, the weight decay rate is set to 0.001, and the L2 regularization value is set to 0.0001, respectively. Each model is trained for 50 epochs, with a batch size of 32 and a shuffle after each epoch. The best-validated model configuration is chosen based on an examination of the training results using different sets of hyperparameters.

### 4.3. Evaluation Metrics

The trained network is assessed based on the 15% of test images reserved for testing. The trained model generates segmented binary masks for the test images, which are then compared to the ground truth binary mask for the test images. The proposed model’s performance is evaluated and compared to that of other segmentation models using the most commonly used evaluation matrices for semantic segmentation, including the dice similarity coefficient (DSC), intersection over union (IoU), accuracy, recall, precision, and specificity [41,42].

## 5. Results

This section presents the findings and experimental outcomes of the LV segmentation. The proposed model’s performance is compared with that of well-known DL segmentation models, Mask R-CNN, FCN, and DeepLab. The effectiveness of these models is investigated using the 15% test images.

### 5.1. Numerical Results

Table 1 displays a comparison of the segmentation performance of the proposed model using the five segmentation evaluation metrics. The table displays the DSC, IoU, accuracy, recall, and specificity average values for all test images. On test images, the proposed model obtains a DSC of 0.0.8637, an IoU of 0.9999, an accuracy of 0.9740, a recall value of 0.9886, and a specificity of 0.8352. Although DeepLab takes less time than other models, as was already determined, its overall performance is the lowest of all models. Due to its two-stage design, the Mask RCNN outperformed the FCN and DeepLab; however, among all segmentation architectures, the proposed model with a single stage had the best results.

Similarly, time spent on both training and segmentation of the LV from test images by the trained model is quantified for each model to evaluate the quickness of the models. Table 2 represents the timing information of the proposed model and other well-known segmentation models. From the data in, Table 2 it is clear that the proposed model not only required less time to train but was also fast enough to successfully segment the LV from the test images. The proposed model took 24.86 s to segment all 900 images or about 0.028 s per image. When compared to Mask RCNN and FCN, the proposed model’s training and testing times are significantly shorter. In contrast, DeepLab’s training and testing times are shorter, but its performance is not competitive with the benchmark.

### 5.2. Visual Results

**Figure 5:**
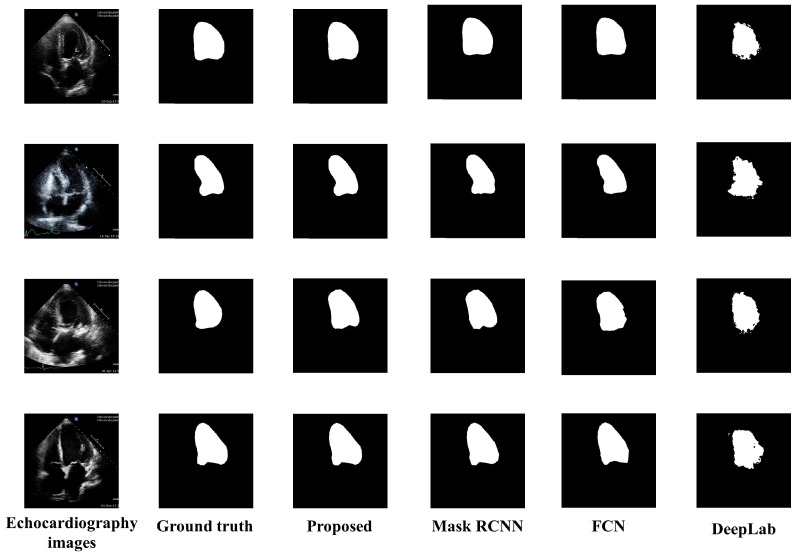
Visual comparisons of the proposed model to various segmentation models.

Figure 5 presents the visual results of the segmented output of all models. Original echocardiography images are in the leftmost column, and the ground truth labels are next to them. The segmented outputs of the proposed model, Mask RCNN, FCN, and DeepLab, are also depicted in column 3, column 4, column 5, and column 6 of Figure 5 respectively. DeepLab segmentation accuracy is not good enough, especially since the borderlines are inaccurate. The Mask RCNN and FCN border lines are quite precise; however, the shape of LV is not as accurate as the proposed model. The proposed model provided a highly accurate representation of the LV’s form as well as its correct contour.

## 6. Discussion

In this work, a lightweight segmentation model is suggested, and its performance, as well as its training and testing times, are compared to DeepLab, FCN, and Mask RCNN. As can be seen in, Table 1 the suggested model achieved the highest levels of performance in terms of DSC, IoU, accuracy, recall, precision, and specificity. The proposed approach extracted features using the RestNet 50 model and then individually processed spatial and channel characteristics to maximize the usage of extracted features and information. On the other hand, the suggested model required much less time to train and segment test images compared to both FCN and Mask RCNN. This is because the spatial and channel information is processed in parallel by the proposed model once the features are extracted from the backbone model.

**Figure 6:**
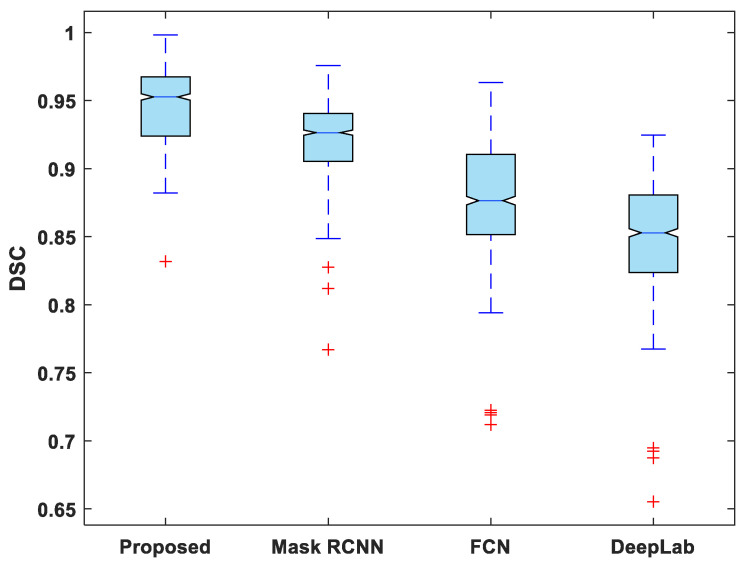
Boxplot of DSC.

In addition to this, the statistical analysis of the evaluation metrics is also presented. A boxplot was used to analyze and compare the distribution of evaluation metric values for all test images. Figure 6 depicts a boxplot of DSC values for test images. The proposed model outperforms competing segmentation methods in terms of the minimum and maximum value, the median, and the upper and lower quartiles. Similarly, the suggested model exhibits fewer outliers than the other models, indicating that its performance is consistent over the entire test dataset.

**Figure 7:**
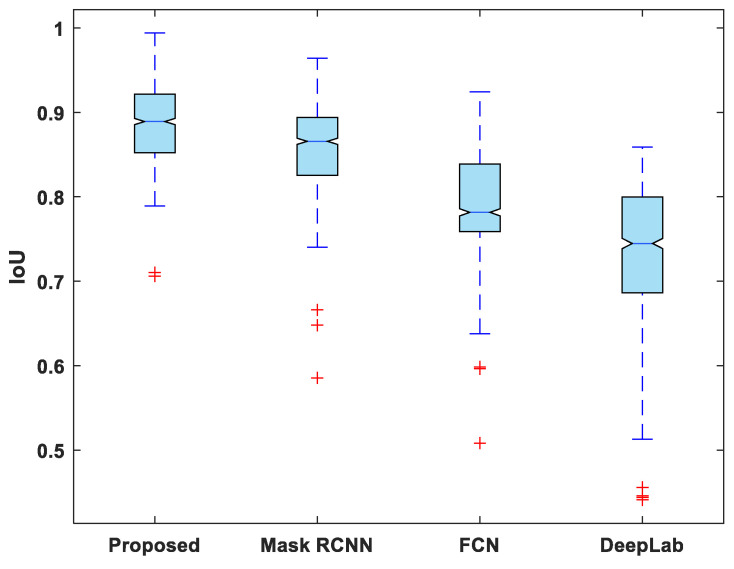
Boxplot of IoU.

**Figure 8:**
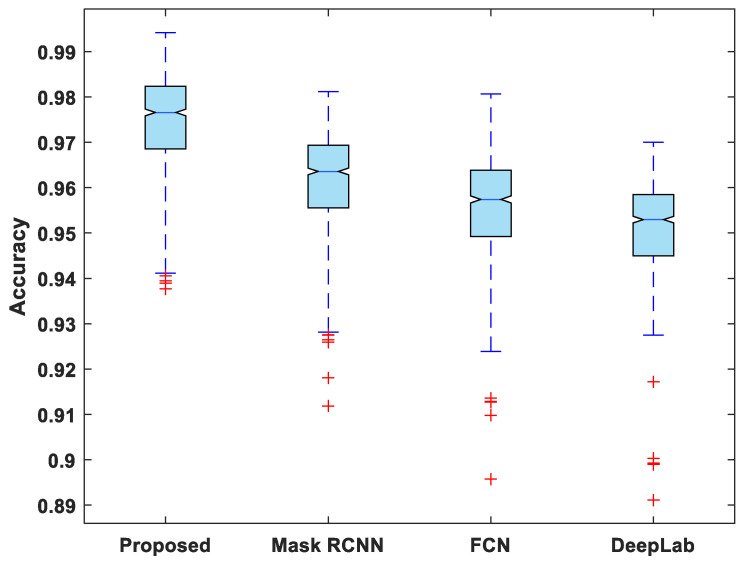
Boxplot of accuracy.

**Figure 9:**
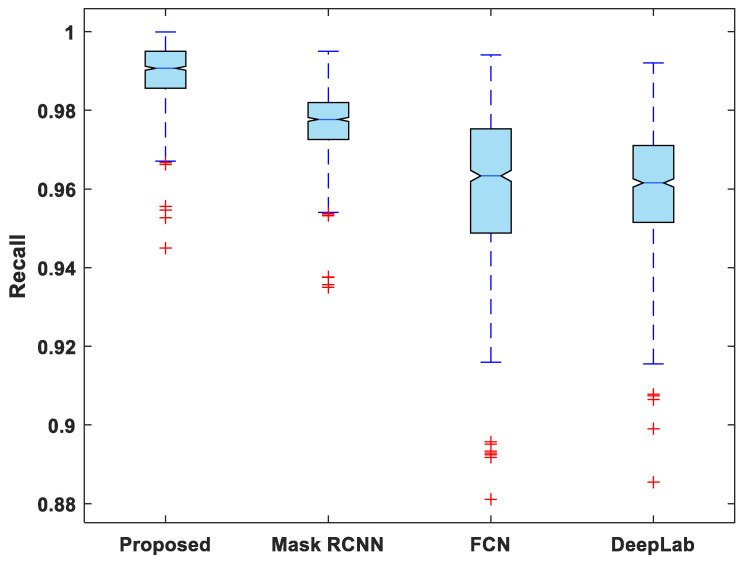
Boxplot of recall.

**Figure 10:**
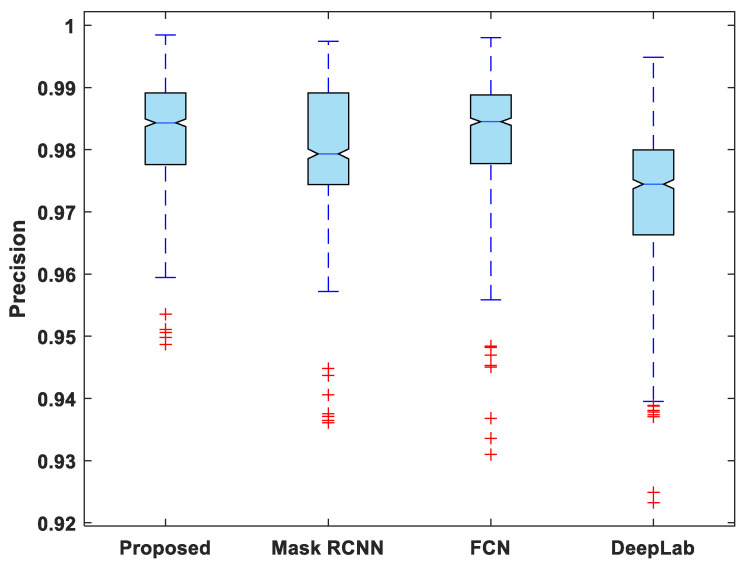
Boxplot of precision.

**Figure 11:**
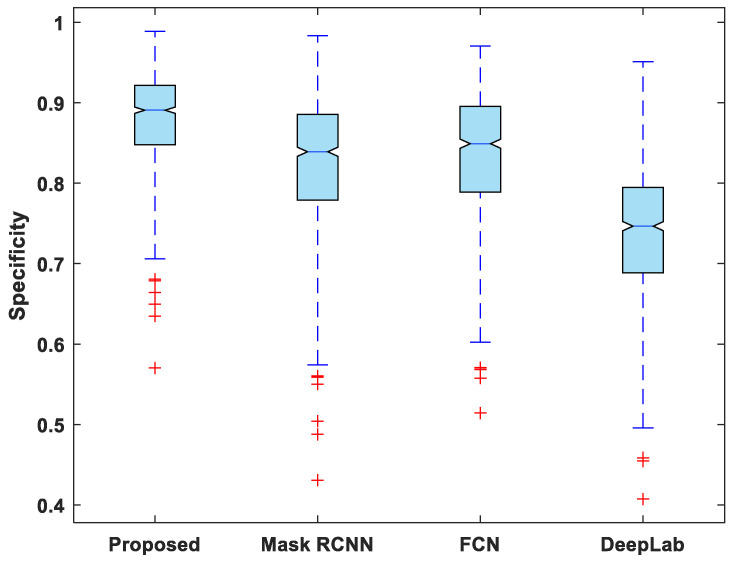
Boxplot of specificity.

Figure 7, Figure 8, Figure 9, Figure 10 and Figure 11 display boxplots for IoU, accuracy, recall, precision, and specificity, respectively, from which it can be inferred that the behavior of the proposed model for these metrics is fairly similar to that of DSC. Furthermore, the proposed model’s higher performance is also demonstrated by the lower skewness of the boxplot compared to the skewness of boxplots for the other models.

## 7. Conclusions

A lightweight segmentation network was designed and applied in this study for automatic LV segmentation from echocardiography images. Utilizing restnet50 as the backbone model, a feature pyramid network is used to extract low and high-level features. The SFU uses pooling, convolution, batch normalization, and sigmoid processes to improve the spatial features generated by the backbone model. Similarly, the channel information is refined using global pooling and multi-layer perceptron in the channel feature unit. Using the feature integration unit, these two different level features are combined. The suggested model performed well not only in terms of segmentation accuracy but also in terms of speed. The segmenting of the test images and training of the model took less time. When compared to the well-known segmentation models Mask RCNN, FCN, and DeepLab, the proposed model scored the highest evaluation metrics with a DSC of 0.9446, IoU of 0.8445, an accuracy of 0.9742, recall of 0.9889, precision of 0.9828, and specificity of 0.8357.

## Figures and Tables

**Table 1:** Mean and standard deviation values of evaluation metrics.

Model	DSC	IoU	Accuracy	Recall	Specificity	Precision
Proposed	0.9446 ±0.0319	0.8845 ±0.0536	0.9742 ±0.0114	0.9889 ±0.0081	0.8357 ±0.0817	0.9828 ±0.0088
Mask RCNN	0.9239 ±0.0232	0.8651 ±0.0457	0.9612 ±0.0116	0.9760 ±0.0084	0.8252 ±0.0845	0.9806 ±0.0087
FCN	0.8758 ±0.0439	0.7978 ±0.0536	0.9560 ±0.0115	0.9617 ±0.0178	0.8468 ±0.0587	0.9818 ±0.0095
DeepLab	0.8485 ±0.0409	0.7448 ±0.0620	0.9513 ±0.0092	0.9593 ±0.0167	0.7403 ±0.0787	0.9728 ±0.0098

**Table 2:** Training and testing time of the models.

Model	Training Time	Segmentation Time of Test Images
Proposed	9 h. 53 min	24.86 s
Mask RCNN	12 h. 37 min	41.27 s
FCN	10 h. 57 min	31.97 s
DeepLab	6 h. 42 min	20.82 s

## Data Availability

All the data are available upon request from the corresponding author.

## Abbreviations

Cardiovascular diseases (CVDs), left ventricle (LV), Deep learning (DL), Fully convolutional networks (FCN), convolutional neural networks (CNNs), spatial feature unit (SFU), channel features unit (CFU), feature integration unit (FIU), dice similarity coefficient (DSC), intersection over union (IoU).
